# Rhinovirus prevalence as indicator for efficacy of measures against SARS-CoV-2

**DOI:** 10.1186/s12889-021-11178-w

**Published:** 2021-06-21

**Authors:** Simo Kitanovski, Gibran Horemheb-Rubio, Ortwin Adams, Barbara Gärtner, Thomas Lengauer, Daniel Hoffmann, Rolf Kaiser, Respiratory Virus Network

**Affiliations:** 1 Bioinformatics and Computational Biophysics, Faculty of Biology and Centre for Medical Biotechnology (ZMB), University of Duisburg-Essen, Essen, 45141 Germany; 2 Institute of Virology, University of Cologne, Faculty of Medicine and University Hospital of Cologne, Cologne, 50935 Germany; 3 Department of Infectious Diseases, Instituto Nacional de Ciencias Médicas y Nutrición Salvador Zubirán, Mexico City, 14080 Mexico; 4 Institute of Virology, University Hospital Düsseldorf, Heinrich Heine University Düsseldorf, Düsseldorf, 40225 Germany; 5 Institute of Medical Microbiology and Hygiene, Saarland University Medical Center, Homburg, 66421 Germany; 6 Computational Biology, Max Planck Institute for Informatics, Saarland Informatics Campus, Saarbrücken, 66123 Germany

**Keywords:** SARS-CoV-2, COVID-19, Rhinovirus, Bayesian, Modeling, Germany

## Abstract

**Background:**

Non-pharmaceutical measures to control the spread of severe acute respiratory syndrome coronavirus 2 (SARS-CoV-2) should be carefully tuned as they can impose a heavy social and economic burden. To quantify and possibly tune the efficacy of these anti-SARS-CoV-2 measures, we have devised indicators based on the abundant historic and current prevalence data from other respiratory viruses.

**Methods:**

We obtained incidence data of 17 respiratory viruses from hospitalized patients and outpatients collected by 37 clinics and laboratories between 2010-2020 in Germany. With a probabilistic model for Bayes inference we quantified prevalence changes of the different viruses between months in the pre-pandemic period 2010-2019 and the corresponding months in 2020, the year of the pandemic with noninvasive measures of various degrees of stringency.

**Results:**

We discovered remarkable reductions *δ* in rhinovirus (RV) prevalence by about 25% (95% highest density interval (HDI) [−0.35,−0.15]) in the months after the measures against SARS-CoV-2 were introduced in Germany. In the months after the measures began to ease, RV prevalence increased to low pre-pandemic levels, e.g. in August 2020 *δ*=−0.14 (95% HDI [−0.28,0.12]).

**Conclusions:**

RV prevalence is negatively correlated with the stringency of anti-SARS-CoV-2 measures with only a short time delay. This result suggests that RV prevalence could possibly be an indicator for the efficiency for these measures. As RV is ubiquitous at higher prevalence than SARS-CoV-2 or other emerging respiratory viruses, it could reflect the efficacy of noninvasive measures better than such emerging viruses themselves with their unevenly spreading clusters.

**Supplementary Information:**

The online version contains supplementary material available at (10.1186/s12889-021-11178-w).

## Background

The Coronavirus Disease 2019 (COVID-19) pandemic is caused by the severe acute respiratory syndrome coronavirus (SARS-CoV-2) [1]. SARS-CoV-2 is transmitted person-to-person predominantly via respiratory droplets and aerosols produced by breathing, coughing or sneezing. These particles are deposited directly on mucosal surfaces, or on fomites [2–4]. Recent evidence suggests that SARS-CoV-2 is quite resilient and may remain infectious in aerosols for hours and on surfaces for days [5]. With no effective treatment or vaccine available, the containment of SARS-CoV-2 depends on measures such as physical distancing, restrictions on mobility, increased personal hygiene, and use of face masks [6–9]. These measures appear to be effective [10] but they also exert substantial social and economic burden [11]. Therefore, governments should tune these measures to limit the spread of SARS-CoV-2, while also allowing a maximum degree of normalcy. To this end, we need reliable indicators for the efficacy of the measures against SARS-CoV-2.

Rigorous measures against SARS-CoV-2 will most likely slow down the spread of the virus, as we have seen in the first COVID-19 wave in different countries around the world. Thus, for the weeks and months after the measures are introduced we expect a reduction in the relative frequency (prevalence) of individuals who test positive for SARS-CoV-2. As the measures against SARS-CoV-2 begin to be relaxed to a point where they are no longer effective in containing the virus, we anticipate resurgence in the prevalence of SARS-CoV-2. This implies that we may use the information about changes in SARS-CoV-2 prevalence over time to assess the efficacy of the measures.

With SARS-CoV-2 being a novel virus, the use of SARS-CoV-2 prevalence as indicator for efficacy of the measures comes with a number of caveats. For instance, due to lack of comparable epidemiological data for this virus from the previous years we have limited understanding of its seasonal variation in transmission, which is likely to exert a strong influence on the dynamics of the pandemic, as we know from other respiratory viruses. An example is provided by the influenza virus: in Europe we observe high influenza prevalence between December and April [12]. For the remaining months of the year, however, the prevalence of influenza is negligible. The lack of historic prevalence data for SARS-CoV-2 means that we cannot disentangle effects of measures from potential seasonal effects. Thus, by ignoring the seasonal variation in transmission, we may under- or overestimate SARS-CoV-2 prevalence, and as a result misjudge the efficacy of measures. Moreover, accurate assessment of the SARS-CoV-2 prevalence depends on robust infrastructure (e.g. testing laboratories, experts, kits, digital platforms for sharing of SARS-CoV-2 data) for rapid detection and reporting of SARS-CoV-2 infections, including widespread screening for asymptomatic SARS-CoV-2 infections [13]. As of yet, such infrastructure is lacking across many parts of the globe, partially blinding us.

In the study presented here, we describe an indirect yet more robust approach for quantifying the efficacy of the measures against SARS-CoV-2. Our core assumption is that efficient measures against SARS-CoV-2 will also suppress the spread of other respiratory viruses that have similar features as SARS-CoV-2, such as transmission routes, viability in different environments, etc. To test this hypothesis, we obtained epidemiological data on the incidences of 17 different respiratory viruses spanning the years 2010-2020. Using a probabilistic model, we inferred the monthly prevalence of these viruses in the pre-pandemic period 2010-2019, and then compared this with the prevalence of the same virus in the months of 2020, i.e., the period following the introduction of the measures against SARS-CoV-2. Thus, we were able to determine to which degree the prevalence of the different respiratory viruses is affected by the measures against SARS-CoV-2, while properly accounting for seasonal effects. Strong deviations in viral prevalence are interpreted and discussed here in the context of the features of the different viruses. A key finding of this study is that rhinovirus prevalence is a suitable indicator for the effectiveness of public health measures against SARS-CoV-2.

## Methods

### Virus prevalence data

Incidence data on 17 different respiratory viruses in hospitalized patients was obtained from the Respiratory Viruses Network (RespVir) [14]. The data was collected from 37 clinics and laboratories across Germany in the period from 2010 to 2020 (up to and including October 2020) (Supplementary Section 1). From RespVir we also obtained incidence data on SARS-CoV-2 collected from 14 laboratories across Germany in the period from 24.01.2020 to 27.10.2020 (Supplementary Section 1). From this data we computed frequencies (counts) of positive tests for each virus that originate from a specific laboratory in a given month and year, including the total number of tests made.

### Statistical modeling of monthly prevalence of 17 respiratory viruses

We used data from the years 2010 to 2020 to model the monthly prevalence of different respiratory viruses. For laboratory *l*∈{1,…,37}, month *m*∈{1,…,12} and year *y*∈{2010,…,2020}, we observed $Y^{v}_{lmy}$ positive cases of the virus *v* among $N^{v}_{{lmy}}$ tested patients. We set the design variable $X^{v}_{{my}}$ 0 for months in the pre-pandemic period (years 2010 to 2019) and 1 for months in 2020. That is, the $X^{v}_{{my}}$ are indicator variables for whether we are in the pandemic year or in a prepandemic year.

We aimed at inferring from these data the mean prevalence of a given virus in each month of the year in the pre-pandemic and pandemic period. Additionally, we aimed at quantifying the effect of the anti-SARS-CoV-2 measures on the prevalence in the different months of the year 2020. For this purpose we designed a likelihood model *M* for Bayesian inference. *M* describes the positive case count as a binomial model:

1 $$\begin{array}{*{20}l} p\left(\left.Y^{v}_{{lmy}}\right|M\right) = \text{Binomial}\left(\pi^{v}_{{lmy}}, N^{v}_{{lmy}}\right), \end{array} $$

where *π* is the probability of positive tests, defined as the inverse-logit function of $\hat {\pi }$:

2 $$\begin{array}{*{20}l} \pi^{v}_{{lmy}} = \mathrm{logit^{-1}}\left(\hat{\pi}^{v}_{{lmy}}\right), \end{array} $$

where logit^−1^(*x*)=1/(1+exp(−*x*)). The raw prevalence varied substantially between individual laboratories. To account for this source of variation, the model treats the corresponding coefficients $\hat {\pi }$ as random samples drawn from a normal distribution:

3 $$\begin{array}{*{20}l} \hat{\pi}^{v}_{{lmy}} \sim \text{Normal}\left(\alpha^{v}_{{my}}, \tau^{v}\right), \end{array} $$

where the inverse-logit of $\alpha ^{v}_{{my}}$ is the mean prevalence of virus *v* in month *m* of year *y*, and *τ*^*v*^ is the standard deviation of virus *v* that accounts for the variance in prevalence between the individual laboratories.

Empirically, we know that the prevalence of most respiratory viruses follows specific seasonal patterns. Hence, the viral prevalence in a given month of the year is not completely independent across the different years in the period from 2010 to 2020. Hierarchical models, such as *M*, enable sharing of information between parameters across the different years by partial pooling [15]. For a specific virus and month, the corresponding coefficients *α* are treated as random samples drawn from a population of parameters:

4 $$\begin{array}{*{20}l} \alpha^{v}_{{my}} \sim \text{Normal}\left(\mu^{v}_{m} + \beta^{v}_{m}X^{v}_{{my}}, \sigma_{m}^{v}\right) \end{array} $$

With a Bayesian approach we can then infer shared parameters for month *m* and virus *v*, such as the mean pre-pandemic prevalence $\text {logit}^{-1}\left (\mu ^{v}_{m}\right)$, the mean pandemic prevalence $\text {logit}^{-1}\left (\mu ^{v}_{m} + \beta ^{v}_{m}\right)$, where coefficient $\beta ^{v}_{m}$ is the effect of the anti-SARS-CoV-2 measures; and the standard deviation $\sigma _{m}^{v}$, while simultaneously accounting for the within-year variability. The weakly informative priors assigned to *β*,*μ* and *σ* and *τ* are defined by:

5 $$\begin{array}{*{20}l} \beta^{v}_{m} \sim \text{Normal}(\mu=0, \sigma=10) \end{array} $$

6 $$\begin{array}{*{20}l} \mu^{v}_{m} \sim \text{Normal}(\mu=0, \sigma=10) \end{array} $$

7 $$\begin{array}{*{20}l} \sigma_{m}^{v} \sim \text{Cauchy}^{+}\,(\mu=0, \gamma=1) \end{array} $$

8 $$\begin{array}{*{20}l} \tau^{v} \sim \text{Cauchy}^{+}\,(\mu=0, \gamma=1) \end{array} $$

For virus *v* and month *m*, we estimate the change in mean prevalence (*δ*) between the pandemic and the pre-pandemic period as:

9 $$\begin{array}{*{20}l} \delta_{m}^{v}=\text{logit}^{-1}\left(\mu^{v}_{m} + \beta^{v}_{m}\right) - \text{logit}^{-1}\left(\mu^{v}_{m}\right) \end{array} $$

For months where *δ*<0 and the 95% Highest Density Intervals (HDIs) of *δ* lie mostly or completely below 0 (0 = null effect), we have strong evidence of reduced viral prevalence in the year 2020 compared to that in the years 2010 to 2019. On the other hand, for months where *δ*>0 and the 95% HDI of *δ* lie mostly or completely above 0, we have strong evidence of increased viral prevalence. Distributions with the 95% HDIs more or less centered around 0 indicate that there is no evidence for a clear change in the monthly viral prevalence in the year 2020. Note that unclear evidence is not equivalent to no change, because for a month with *δ*≈0 we may also have a wide 95% HDI, including possibilities for positive or negative change.

Notably, the inference of each coefficient *β* relies on only one small data set (one value per contributing lab) for each month of year 2020. Therefore, the *β* coefficients will be highly uncertain and have wide 95% HDIs. Consequently, the coefficients *δ* will also be uncertain.

*M* was implemented in Stan [16]. Inference of the parameters of *M* was executed with rstan using the No-U-Turn sampler by running a Markov chain Monte Carlo (MCMC) simulation with six chains of 10,000 iterations each, including 3,000 warm-ups (R-package rstan, version 2.19.2). To test the validity of our model, we performed posterior predictive checks. We used the potential scale reduction factor (PSRF), the effective number of samples (*N*_*eff*_) and information provided by rstan on divergences during the MCMC sampling to check for a successful convergence. For each parameter we report its posterior median and 95% HDI.

### Statistical modeling of monthly SARS-CoV-2 prevalence

For laboratory *l*∈{1,…,14} and month *m*∈{1,…,10} in 2020, we observe *Y*_*lm*_ positive cases of SARS-CoV-2 among *N*_*lm*_ tested patients. From this data we infer the overall monthly prevalence of positive cases. To this end we designed a likelihood model *M*_*SC*2_ for Bayesian inference. With *M*_*SC*2_ we describe the count of positive SARS-CoV-2 cases as a binomial model:

10 $$\begin{array}{*{20}l} p(Y_{{lm}}|M_{{SC2}}) = \text{Binomial}(\pi_{{lm}}, N_{{lm}}), \end{array} $$

where *π* is the probability of positive tests, defined as the inverse-logit of *α*:

11 $$\begin{array}{*{20}l} \pi_{{lm}} = \mathrm{logit^{-1}}\left(\alpha_{{lm}}\right) \end{array} $$

The SARS-CoV-2 prevalence varied substantially between individual laboratories. To account for this source of variation, the model treats the corresponding coefficients *α* as random samples drawn from a population of parameters:

12 $$\begin{array}{*{20}l} \alpha_{{lm}} \sim \text{Normal}(\mu_{m}, \sigma), \end{array} $$

where *μ*_*m*_ is the mean SARS-CoV-2 prevalence for a specific month *m*, and *σ* is the standard deviation. The weakly informative priors assigned to *μ*_*m*_ and *σ* are defined by:

13 $$\begin{array}{*{20}l} \mu_{m} \sim \text{Normal}(\mu=0, \sigma=10) \end{array} $$

14 $$\begin{array}{*{20}l} \sigma \sim \text{Cauchy}^{+}\,(\mu=0, \gamma=1) \end{array} $$

*M*_*SC*2_ was implemented in Stan and executed with MCMC simulation settings identical to those introduced for model *M*. For each parameter we report its posterior median and 95% HDI.

With *M*_*SC*2_ we can also infer the mean SARS-CoV-2 prevalence in different weeks of the year based on the RespVir data on SARS-CoV-2 frequencies in different lab- oratories and weeks of year 2020 (Supplementary FigureS2). For this analysis we have to substitute the month-specific index *m*∈{1,…,10} with the week-specific index *m*∈{4,…,43} in *M*_*SC*2_.

## Results

### SARS-CoV-2 prevalence in 2020

**Fig. 1:**
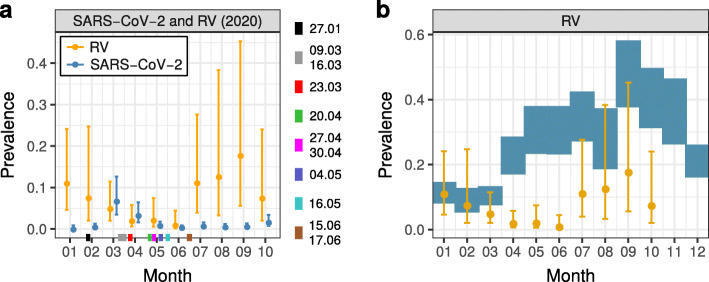
Monthly prevalence of SARS-CoV-2 and RV. **a** Blue circles and bars: mean SARS-CoV-2 prevalence between January 2020 and October 2020 with the corresponding 95% HDIs. Orange circles and bars: mean pandemic RV prevalence between January 2020 and October 2020 with the corresponding 95% HDIs. Colored rectangles along the x-axis at dates of specific measures, relaxations or other important events in 2020 [17]. 27.01: first confirmed case of SARS-CoV-2 in Germany; 09.03: large events are canceled; 16.03: schools, child care, shops, churches, bars, etc. are closed; 23.03: contact ban; 20.04: shops (partially) reopen; 27.04: mandatory use of face masks; 30.04: museums, temples, zoos and playgrounds reopen; 04.05: schools (partially) reopen; 16.05: restaurants reopen; 15.06: European Union and Schengen countries reopen borders; 17.06: more than 1,000 meat-factory workers test positive for SARS-CoV-2. **b** Blue rectangles: 95% HDIs of the mean pre-pandemic RV prevalence in each month of the year. Orange circles and bars refer to RV as in panel (**a**)

We evaluated the monthly SARS-CoV-2 prevalence in 2020 (Fig. 1A). For January 2020 our data does not contain positive cases for SARS-CoV-2. Hence, the mean SARS-CoV-2 prevalence was 0 [0,0.009] (in the following we give the 95% HDIs behind numbers in square brackets to quantify uncertainty). In February 2020 a few of our laboratories found SARS-CoV-2 (SupplementaryFigure S1), however, the overall mean SARS-CoV-2 prevalence remained low (0.005 [0.002,0.013]). SARS-CoV-2 prevalence reached its peak at 0.067 [0.035,0.126] in March 2020. Around mid-March 2020, the initial set of measures against SARS-CoV-2 was introduced in Germany [17]. This was followed by a drop in mean SARS-CoV-2 prevalence to 0.032 [0.016,0.066] in April 2020 and to 0.009 [0.004,0.018] in May 2020. Between June 2020 and September 2020, the SARS-CoV-2 prevalence remained approximately flat. In June, July, August and September 2020, the mean SARS-CoV-2 prevalence was 0.004 [0.002,0.009],0.007 [0.003,0.016],0.005 [0.002,0.012] and 0.006 [0.003,0.014], respectively. In October 2020 new SARS-CoV-2 cases surged again [18], increasing the mean SARS-CoV-2 prevalence to 0.016 [0.007,0.035] (Fig. 1A).

### Among 17 respiratory viruses, rhinovirus is most strongly affected by anti-SARS-CoV-2 measures

**Fig. 2:**
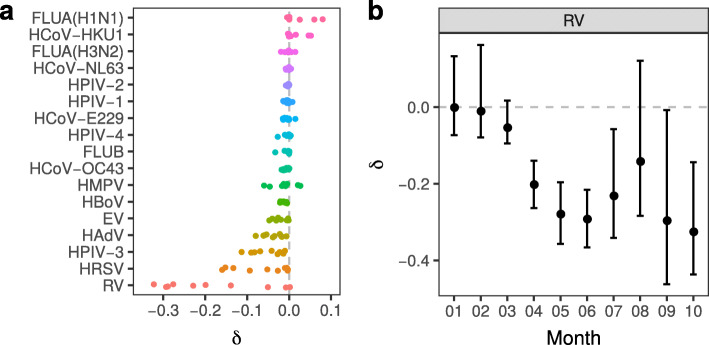
Change in mean monthly prevalence (*δ*) of different respiratory viruses between the pandemic (2020) and pre-pandemic (2010-2019) period. **a** Colored circles: median coefficients *δ* for different months of the year (x-axis) and different respiratory viruses (y-axis). Random vertical jitter was added to avoid overplotting. **b** Change in mean RV prevalence. Black circles and bars: median coefficients *δ* with the corresponding 95% HDIs. Dashed lines at *δ*=0

As a result of the measures against SARS-CoV-2, we also expect a reduction in the spread of other respiratory viruses that have similar features as SARS-CoV-2. To test this hypothesis, we compared the monthly prevalence of 17 different respiratory viruses between the years 2010-2019 (pre-pandemic period) and 2020 (pandemic period) in Germany (Supplementary Figure S3). For each month of the year we report the change in mean prevalence (*δ*) of each respiratory virus between the year 2020 and the period 2010-2019 (Supplementary Figure S4). For almost all respiratory viruses, *δ* values are predominantly negative, i.e. these viruses have lower prevalence in 2020 (Fig. 2A).

For rhinovirus (RV) we observed exceptionally strong suppression of its prevalence during that period (Fig. 2A/B). Other viruses, such as human respiratory syncytial virus (HRSV), human parainfluenza virus 3 (HPIV-3), human adenovirus (HAdV) and enterovirus (EV), decreased moderately from April 2020 to October 2020, compared to the respective months from 2010 to 2019 (Fig. 2A, Supplementary Figure S3). For several other respiratory viruses (human bocavirus (HBoV) to influenza A virus subtype H3N2 (FLUA(H3N2)) in Fig. 2A) there was a small to negligible trend to lower *δ*, and only 2 of the 17 viruses, human coronavirus HKU1 (HCoV-HKU1) and influenza A virus subtype H1N1 (FLUA(H1N1)), had a positive *δ* trend.

Virus seasonality is one determinant of the degree to which the prevalence of different respiratory viruses reflects the measures against SARS-CoV-2. RV and several of the other viruses (HAdV, HPIV-3) whose prevalence are moderately suppressed by the measures, are continuously present in the general population at a prevalence of a few percent (Supplementary Figure S3). Only this high baseline prevalence allowed us to detect moderate or strong reduction in their prevalence in the period from April 2020 to October 2020. HRSV prevalence, on the other hand, varies strongly between different seasons, i.e. during winter and early spring we observe high HRSV prevalence in Europe, and low prevalence during the rest of the year [12]. Thus, we were able to detect moderate reduction in HRSV prevalence from April 2020 to June 2020 when HRSV prevalence was still sufficiently high, but not for July to October 2020 when HRSV has a low prevalence anyway.

The remaining respiratory viruses either have transmission patterns that vary drastically between different seasons, or have low prevalence throughout the year (Sup-plementary Figure S5). In either case, these viruses were barely present in Germany in the period from April 2020 to October 2020, so that changes in their prevalence in that period due to anti-SARS-CoV-2 measures could not be detected reliably.

### Rhinovirus prevalence

In the above analysis, rhinovirus (RV) had particularly strong changes in prevalence between April and October 2020, which made it most promising as an indicator of efficacy of anti-SARS-CoV-2 measures. Hence, we focus on RV in the following.

First, in January 2020 and February 2020, the months before the anti-SARS-CoV-2 measures were introduced in Germany, the mean RV prevalence (orange circles and bars in Fig. 1B) is consistent with the pre-pandemic mean RV prevalence (blue rectangles in Fig. 1B). The mean RV prevalence in March 2020 is slightly lower yet still has large overlap with the mean pre-pandemic RV prevalence (Fig. 1B).

Second, between April 2020 and June 2020, the mean RV prevalence falls completely outside the 95% HDI of the pre-pandemic mean RV prevalence for the respective months (Fig. 1B). The drastic reduction in mean RV prevalence in April 2020 (*δ*=−0.2 [−0.26,−0.14]), May 2020 (*δ*=−0.28 [−0.36,−0.2]) and June 2020 (*δ* = −0.29 [−0.37,−0.22]) may be attributed to the measures against SARS-CoV-2. In July 2020 (*δ* = −0.23 [−0.34,−0.06]), August 2020 (*δ*=−0.14 [−0.28,0.12]) and September 2020 (*δ*=−0.29 [−0.46,−0.01]) we observed a moderate resurgence in mean RV prevalence to low pre-pandemic levels. In October 2020 (*δ*=−0.32 [−0.44,−0.14]) the mean RV prevalence once again falls completely outside the 95% HDI of the pre-pandemic mean RV prevalence for the respective month (Fig. 1B).

Third, while from 2010 to 2019 the RV prevalence exhibited a clear seasonal upward trend from February to June (upward shift of blue rectangles in Fig. 1B), the data for 2020 shows an unbroken downward trend from February to June (orange circles in Fig. 1A/B), contrary to the seasonal trend. It seems that these dynamics in 2020 could have started even before the implementation of anti-SARS-CoV-2 measures in mid-March, possibly because many individuals, alerted by the intensive news coverage, have changed their behavior.

## Discussion

### Transmission properties of RV and SARS-CoV-2 explain their dynamics during the pandemic

To contain the spread of SARS-CoV-2 in Germany, a package of diverse measures was introduced around mid-March 2020 [17]. With SARS-CoV-2 presumed to spread by both airborne and contact-based pathways, the measures were aimed at reducing the number of person-to-person contacts by e.g. physical distancing and closure of shops and schools, and by more rigorous personal hygiene with e.g. more frequent hand washing, use of face masks, and disinfecting surfaces. Soon after the measures were enacted, we observed a reduction in SARS-CoV-2 prevalence (Fig. 1A). As a byproduct of the measures against SARS-CoV-2, the transmission of other respiratory viruses, such as RV, appears to have been diminished as well (Fig. 2B, Supplementary Figure S4). Between mid-April 2020 and mid-June 2020, gradual relaxation of the measures took place, including partial re-opening of shops and schools, lifting of travel restrictions and reopening of playgrounds and churches [17]. Soon thereafter, namely between July 2020 and September 2020, the RV prevalence increased to low pre-pandemic levels, while the SARS-CoV-2 prevalence remained approximately flat. In October 2020 the number of new SARS-CoV-2 cases in Germany surged, and so did our estimated SARS-CoV-2 prevalence. In contrast to this, the RV prevalence decreased in October 2020, though with a still large uncertainty (Fig. 1B). To better understand why SARS-CoV-2 and RV exhibit similar dynamics between April 2020 and June 2020, and divergent dynamics between July 2020 and October 2020, we examine some of their features more closely.

RV is the most common respiratory pathogen of humans and a major causative agent of the common cold [19, 20]. RV is likely transmitted via respiratory aerosols produced by coughing or sneezing, and by contact with surfaces contaminated with nasal secretions [20]. Owing to its stability, RV may remain infectious on surfaces for days and in aerosols for hours [21, 22]. While the assessment of SARS-CoV-2 transmission and resilience in different environments is still an area of active research, recent studies have reported similar transmission routes and degree of resilience for different human coronaviruses (HCoVs) including for SARS-CoV-2 [5, 22]. If we assume that transmission routes of RV and SARS-CoV-2 have a large overlap, then we can expect that the rigorous anti-SARS-CoV-2 measures will affect the spread of both viruses to a similar degree. This is a plausible explanation for the consistent decrease in RV and SARS-CoV-2 prevalence between April 2020 and June 2020.

However, there are also differences between RV and SARS-CoV-2 that have to be considered, namely differences in (1) seasonal patterns, and (2) the degree of dissemination in the human population.

First, the introduction of anti-SARS-CoV-2 measures have certainly lowered the prevalence of RV and SARS-CoV-2 but even these lower levels are still modulated by seasonal patterns. We know empirically that there is a seasonal upwards trend in RV prevalence from February to September (Fig. 1B). Conversely, there is a downward trend in the prevalence of different HCoVs during summer and autumn [23] (Supplementary Figure S3). If we assume that SARS-CoV-2 follows a seasonal trend that is similar to that of other HCoVs, then the combined effect of the season and of anti-SARS-CoV-2 measures is probably responsible for the flat SARS-CoV-2 prevalence between July and September 2020. In the same vein, the divergence of RV and SARS-CoV-2 prevalence courses in summer 2020 could also be due to seasonal changes that modulate the low level RV and SARS-CoV-2 prevalence still suppressed by anti-SARS-CoV-2 measures. As the activity of HCoVs peaks in the coming months of fall and winter and the activity of RV declines (Fig. 1B), we expect to see a rebound of SARS-CoV-2 prevalence to a level similar to the RV prevalence and again a crossing of the two prevalence curves as observed between February and March 2020. Already in October 2020 we see evidence in support of such dynamics between RV and SARS-CoV-2 (Fig. 1A). As a result of the combined effect of the new anti-SARS-CoV-2 measures introduced in Germany in November 2020 [17], and the seasonal suppression of RV being close to its inflection point, we expect again a roughly parallel decay of RV and SARS-CoV-2 prevalence if the measures are effective.

Second, we know that RV is widespread in the human population and environment [19]. Hence, rapid resurgence in RV prevalence might be possible in response to the relaxations. With SARS-CoV-2 being less widely disseminated within the European population, it is also possible that it takes longer for SARS-CoV-2 to reemerge in response to the relaxations.

In summary, generally low levels of RV prevalence between January and October 2020 are consistent with the effectiveness of anti-SARS-CoV-2 measures in Germany, though we emphasize that seasonality of viral prevalence cannot be neglected. These results are corroborated by reports from the United Kingdom [24] and Australia [25, 26] where after lock-downs low RV prevalence values were observed that then bounced back after the easing of restrictions [27].

### RV prevalence is the most suitable indicator of efficacy for the anti-SARS-CoV-2 measures among all studied viruses

Our study reveals reduced prevalence of the respiratory viruses HRSV, HPIV-3, HAdV and EV between April 2020 and October 2020. HRSV, HAdV and EV are transmitted similarly to RV and SARS-CoV-2, namely via respiratory droplets and aerosols, and direct or indirect contact [21, 28, 29], while limited experimental data for HPIV-3 hints at fomites as its main route of transmission [21]. There are several arguments in favor of using the RV prevalence over the prevalence of the other respiratory viruses as indicator of efficacy for the measures against SARS-CoV-2.

First, RV is constantly circulating in the population and is therefore subject to significantly lower seasonal fluctuations than other respiratory viruses, such as HRSV (Supplementary Figure S3). Second, the prevalence of RV is typically higher throughout the year than the prevalence of HRSV, HPIV-3, HAdV and EV (SupplementaryFigure S3). Hence, larger decreases in prevalence, which are also easier to detect by our approach, are possible for RV as a result of the measures (Fig. 1B). Third, we see that the prevalence of HRSV, HPIV-3, HAdV and EV increases with a longer time delay in response to the relaxations of the anti-SARS-CoV-2 measures in comparison to the RV prevalence (Supplementary Figure S3). These features favor RV prevalence as a quickly responding indicator of anti-SARS-CoV-2 measure efficacy.

In other geographical regions where respiratory viruses exhibit different seasonal patterns of transmission, another respiratory virus might be a more appropriate indicator of the anti-SARS-CoV-2 measure efficacy. For instance, several studies of seasonal influenza virus in Japan [30], South Korea [31], Singapore [32], Australia, Chile, South Africa and the United States [33] have reported suppressed influenza prevalence in the period after the implementation of non-pharmaceutical measures against SARS-CoV-2. Owing to the low seasonal prevalence of influenza in Germany in the period between April 2020 and October 2020, our study shows only a negligible reduction in influenza prevalence (SupplementaryFigure S3).

## Conclusions

Using one virus, such as RV, to monitor measures against another virus, such as SARS-CoV-2, is seemingly paradoxical. However, a mixture of factors such as the high transmissibility of SARS-CoV-2, its initially complex dissemination pattern of exponential growth in many clusters, and the limited testing capacities at the begin of the pandemic, had left us partially blinded regarding the efficacy of anti-viral measures in the first months. In such a situation, the prevalence of RV, a ubiquitous respiratory virus with a long historic record, moderate seasonality and transmission routes similar to SARS-CoV-2, is likely a better indicator of the efficacy of anti-SARS-CoV-2 measures than the prevalence of the latter virus itself. This logic also applies to RV in relation to other respiratory viruses that are candidates for causing future epidemics or pandemics.

## Supplementary Information

**Additional file 1** This file includes Supplementary Section 1 and Supplementary Figure S1–S5.

## Data Availability

Data and source code are available upon reasonable request from Rolf Kaiser and Ortwin Adams (data) and Simo Kitanovski (source code).

## Abbreviations

COVID-19: Coronavirus Disease 2019
EV: enterovirus
FLU: influenza
HAdV: human adenovirus
HBoV: human bocavirus
HCoV: human corona virus
HDI: Highest Density Interval
HMPV: human metapneumovirus
HPIV: human parainfluenza virus
HRSV: human respiratory syncytial virus
MCMC: Markov chain Monte Carlo
*N*_*e**f**f*_: effective number of samples
PSRF: potential scale reduction factor
RespVir: Respiratory Viruses Network
RV: rhinovirus
SARS-CoV-2: severe acute respiratory syndrome coronavirus 2.
