# Supplementary material for: Rhinovirus prevalence as indicator for efficacy of measures against SARS-CoV-2
Source: BMC Public Health. 2021 Jun 21;21:1178. doi: 10.1186/s12889-021-11178-w (PMC8215636; doi:10.1186/s12889-021-11178-w)
Supplement: Supplementary file 1 — Additional file 1 This file includes Supplementary Section 1 and Supplementary Figure S1–S5. [file 12889_2021_11178_MOESM1_ESM.pdf]

Rhinovirus prevalence as indicator for efficacy of measures against SARS-CoV-2 Additional file 1 — Supplementary material

Simo Kitanovski[1†], Gibran Horemheb-Rubio[2,3†], Ortwin Adams[4], Barbara Gärtner[5], Thomas Lengauer[6], Daniel Hoffmann[1*], Rolf Kaiser[2], Respiratory Virus Network

[1]Bioinformatics and Computational Biophysics, Faculty of Biology and Centre for Medical Biotechnology (ZMB), University of Duisburg-Essen, Essen, Germany
[2]Institute of Virology, University of Cologne, Faculty of Medicine and University Hospital of Cologne, Cologne, Germany
[3]Department of Infectious Diseases, Instituto Nacional de Ciencias Médicas y Nutrición Salvador Zubirán, Vasco de Quiroga 15, 14080 Mexico City, Mexico.
[4]Institute of Virology, University Hospital Düsseldorf, Heinrich Heine University Düsseldorf, Düsseldorf, Germany
[5]Institute of Medical Microbiology and Hygiene, Saarland University, Homburg/Saar, Germany
[6]Computational Biology, Max Planck Institute for Informatics, Saarland Informatics Campus, Saarbrücken, Germany

This file includes:
- Supplementary Section 1
- Supplementary Figure S1-S5

Supplementary Section 1: Virus prevalence data

Respiratory virus prevalence data

Incidence data on 17 different respiratory viruses in hospitalized patients and, more recently, outpatients was obtained from the Respiratory Viruses Network (RespVir, https://clinical-virology.net). RespVir performs ongoing active surveillance of different respiratory viruses. While there is no standardized testing protocol for the laboratories contributing their data to RespVir in Germany, the testing is often performed by the use of panels, i.e. tests for multiple respiratory viruses are carried out for each patient. The data contain 1,221,759 tests made in 38 clinics and laboratories across Germany in the period from 2010 to 2020 (up to and including October 2020). The data do not account for co-infections. Importantly, individual tests collected by RespVir are not manually curated. To remove tests that are potentially erroneous we performed post-hoc curation. After consultation with members of the RespVir network, we removed 11,527 tests (less than 1% of all tests) originating from laboratories that report only positive cases of a virus in a given month and year. The final (curated) data contain 1,210,232 tests made in 37 clinics and laboratories across Germany. From this data we computed frequencies (counts) of positive tests for each virus that originate from a specific laboratory in a given month and year, including the total number of tests made.

SARS-CoV-2 prevalence data

From RespVir we obtained incidence data (952,366 tests from 14 laboratories across Germany) on SARS-CoV-2 in the period from 24.01.2020 to 27.10.2020. From these data we computed frequencies (counts) of positive tests for SARS-CoV-2 that originate from a specific laboratory in a given month of the year 2020, including the total number of tests made. Analogously, we computed the frequencies of positive SARS-CoV-2 tests and the total number of tests made in each laboratory and week of the year 2020. We compared the weekly SARS-CoV-2 prevalence data from RespVir with the more extensive data reported by the Robert Koch-Institute (RKI) (downloaded on 28.10.2020 from https://www.rki.de/DE/Content/InfAZ/N/Neuartiges_Coronavirus/Testzahl.html?nn=13490888). We observed similar trends in SARS-CoV-2 prevalence based on the RespVir and RKI data (Supplementary Figure S2).

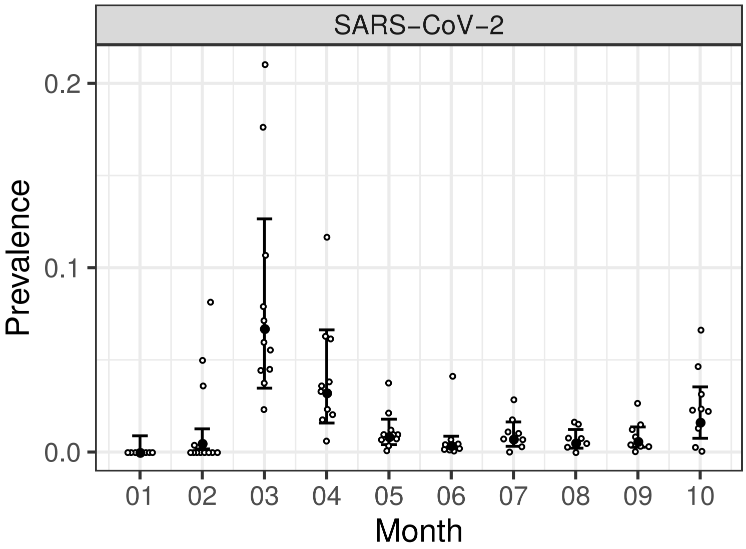

Supplementary Figure S1. SARS-CoV-2 prevalence in different months of the year 2020 in Germany. White circles: SARS-CoV-2 prevalence reported by different laboratories in the months between January 2020 and October 2020 in Germany. Black circles and bars: mean SARS-CoV-2 prevalence in months of year 2020 with the corresponding 95% HDIs. Random jitter along x-axis was added to avoid overplotting.

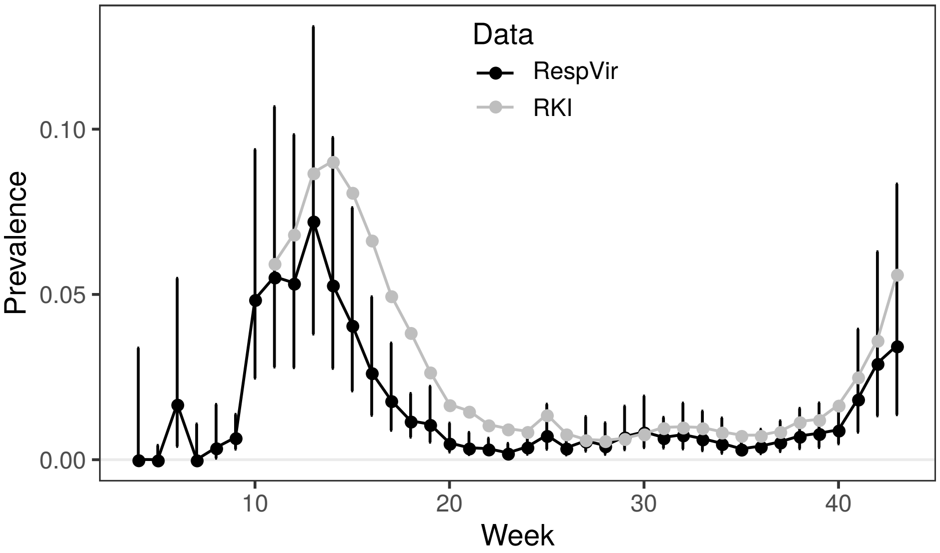

Supplementary Figure S2. SARS-CoV-2 prevalence in different weeks of the year 2020 in Germany. Gray circles: SARS-CoV-2 prevalence between week 11 and week 43 of year 2020 reported by the Robert Koch-Institute (RKI). Black circles and bars: mean SARS-CoV-2 prevalence with the corresponding 95% HDIs between week 4 and week 43 of year 2020, inferred with model M_{SC2} based on data from the Respiratory Viruses Network (RespVir).

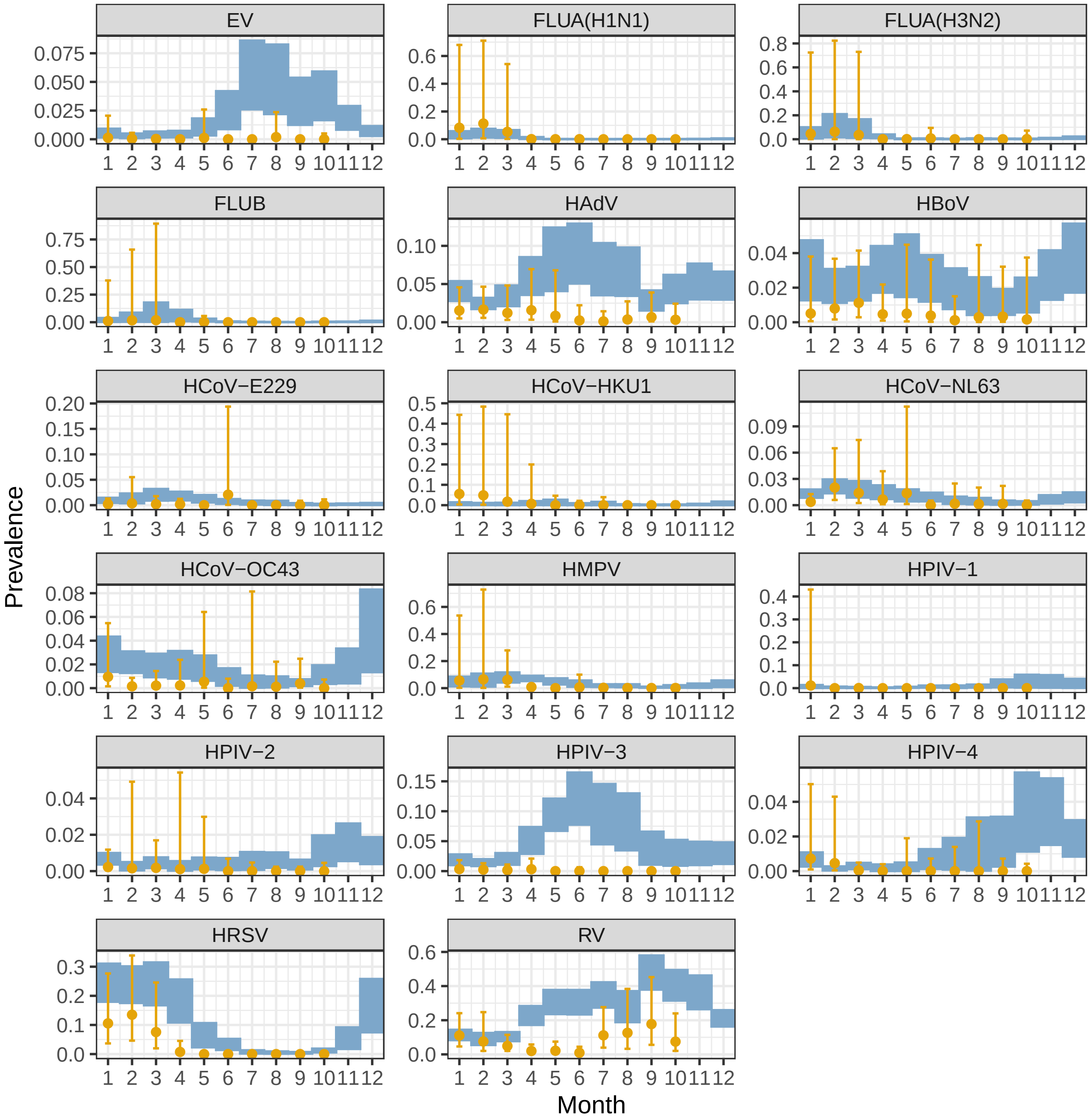

Supplementary Figure S3. Monthly prevalence of 17 respiratory viruses in Germany. Blue rectangles: 95% HDIs of the mean pre-pandemic RV prevalence in each month of the year. Orange circles and bars: mean pandemic RV prevalence between January 2020 and October 2020 with the corresponding 95% HDIs. RV=rhinovirus; HPIV=human parainfluenza virus; HAdV=human adenovirus; HRSV=human respiratory syncytial virus; EV=enterovirus; FLU=influenza; HCoV=human corona virus; HMPV=human metapneumovirus; HBoV=human bocavirus.

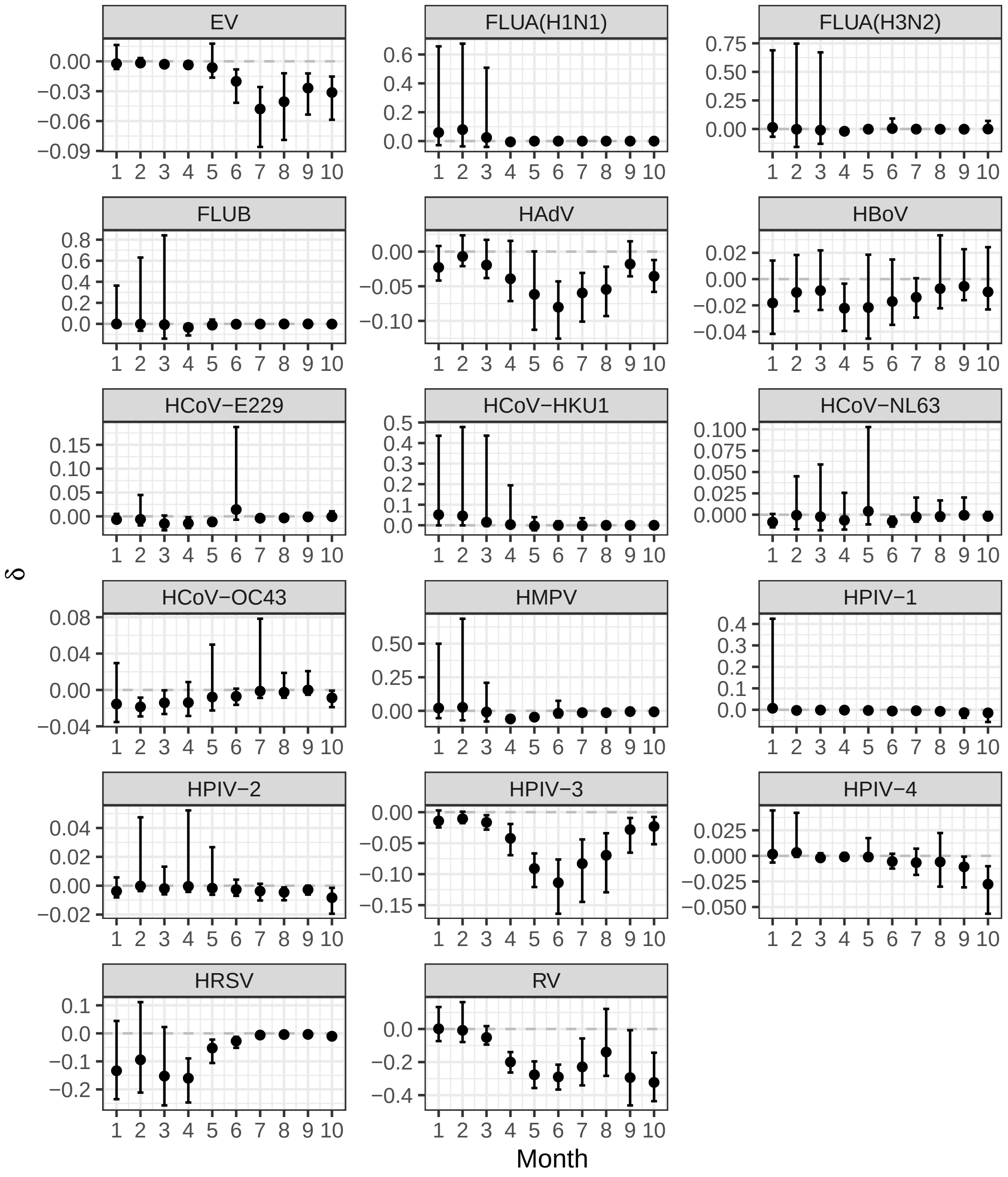

Supplementary Figure S4. Change in mean monthly prevalence (δ) of different respiratory viruses between the pandemic (2020) and pre-pandemic (2010-2019) period. Black circles and bars: median δ with the corresponding 95% HDIs. Horizontal dashed line at δ=0.

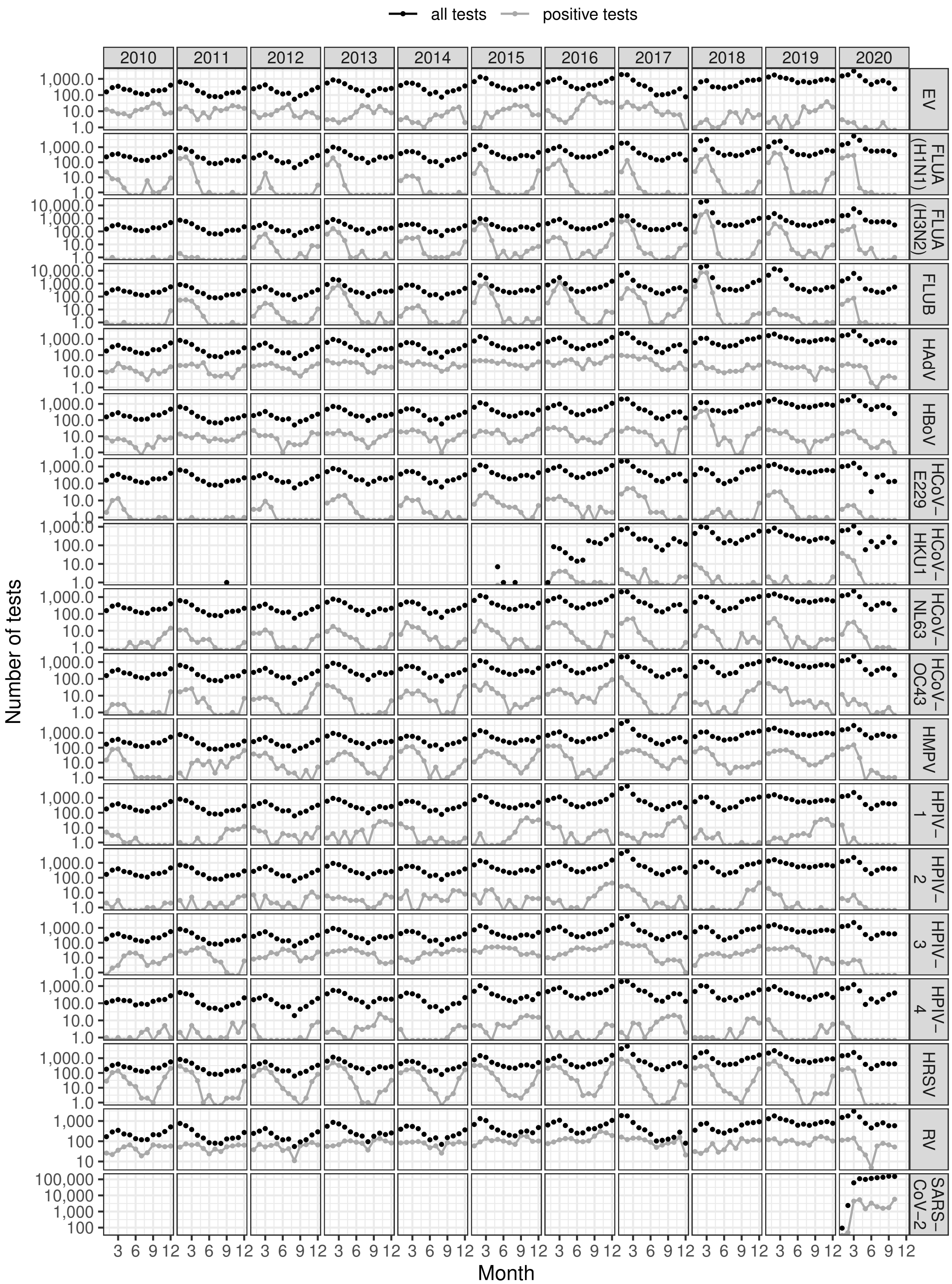

Supplementary Figure S5. Number of positive tests (gray circles connected by lines) and all tests (black circles) for each respiratory virus in different months and years. The y-axis is shown on log10-scale.
